# Structural connectome alterations between individuals with autism and neurotypical controls using feature representation learning

**DOI:** 10.1186/s12993-024-00228-z

**Published:** 2024-01-24

**Authors:** Yurim Jang, Hyoungshin Choi, Seulki Yoo, Hyunjin Park, Bo-yong Park

**Affiliations:** 1 Artificial Intelligence Convergence Research Center, Inha University, Incheon, Republic of Korea; 2 Department of Electrical and Computer Engineering, Sungkyunkwan University, Suwon, Republic of Korea; 3 Center for Neuroscience Imaging Research, Institute for Basic Science, Suwon, Republic of Korea; 4 Convergence Research Institute, Sungkyunkwan University, Suwon, Republic of Korea; 5 School of Electronic and Electrical Engineering, Sungkyunkwan University, Suwon, Republic of Korea; 6 Department of Data Science, Inha University, Incheon, Republic of Korea

**Keywords:** Autism spectrum disorder, Autoencoder, Feature representation learning, Structural connectivity, Integrated gradient

## Abstract

Autism spectrum disorder is one of the most common neurodevelopmental conditions associated with sensory and social communication impairments. Previous neuroimaging studies reported that atypical nodal- or network-level functional brain organization in individuals with autism was associated with autistic behaviors. Although dimensionality reduction techniques have the potential to uncover new biomarkers, the analysis of whole-brain structural connectome abnormalities in a low-dimensional latent space is underinvestigated. In this study, we utilized autoencoder-based feature representation learning for diffusion magnetic resonance imaging-based structural connectivity in 80 individuals with autism and 61 neurotypical controls that passed strict quality controls. We generated low-dimensional latent features using the autoencoder model for each group and adopted an integrated gradient approach to assess the contribution of the input data for predicting latent features during the encoding process. Subsequently, we compared the integrated gradient values between individuals with autism and neurotypical controls and observed differences within the transmodal regions and between the sensory and limbic systems. Finally, we identified significant associations between integrated gradient values and communication abilities in individuals with autism. Our findings provide insights into the whole-brain structural connectome in autism and may help identify potential biomarkers for autistic connectopathy.

**Supplementary Information:**

The online version contains supplementary material available at 10.1186/s12993-024-00228-z.

## Introduction

Autism spectrum disorder is a pervasive condition that occurs during development. Individuals with autism show deficits in sensory processing and social communication skills [1, 2]. To identify the pathological and behavioral associations of autism, previous neuroscience studies have investigated alterations in large-scale functional brain networks [3–5] and abnormalities in microcircuit functions, such as excitation/inhibition imbalances [6–10]. Studies have suggested that autism is associated with altered macroscale functional brain organization, as well as atypical neural circuits and cognitive functions.

Recent neuroimaging studies based on magnetic resonance imaging (MRI) have adopted dimensionality reduction techniques to study high-dimensional connectome data with multiple low-dimensional eigenvectors [11, 12]. The core of these techniques is the generation of low-dimensional features that sufficiently represent whole-brain connectome data by estimating the principal axes of the brain. Functional and microstructural MRI studies have applied dimensionality reduction techniques to connectivity data and observed cortical hierarchical patterns expanding from low-level sensory to higher-order default mode networks [12–14]. Moreover, these techniques have been adopted to investigate structural and functional connectome disorganization in individuals with autism, which consistently suggests altered connectivity in the sensory and default mode regions [3, 5]. In addition to these connectome manifold approaches, feature representation learning based on deep learning techniques is a notable method for generating representative latent features from the original data. In particular, an autoencoder reduces high-dimensional data through multiple nonlinear operations and generates latent vectors in a hidden bottleneck layer. These low-dimensional features are then used to reconstruct the original data [15, 16]. The generated latent features can be used to develop disease diagnosis models; for example, to classify healthy controls and patients with Alzheimer’s disease [17, 18] or schizophrenia [19, 20]. However, one challenge of the autoencoder is the interpretation of latent vectors. To address this issue, a recent study introduced an integrated gradient technique that computes the contribution of the input data to predict features in the hidden layer [21]. Thus, the extent to which the structural connectivity contributes to predicting the low-dimensional latent vectors can be calculated. Here, we hypothesized that the low-dimensional latent features derived from the autoencoder model might contribute differently to the reconstruction of the original connectome data between typically and atypically developing brains.

In this study, we investigated the structural network disorganization in individuals with autism using autoencoder-based feature representation learning. First, we constructed autoencoders to reconstruct the structural connectivity of neurotypical controls and individuals with autism, and generated low-dimensional latent features for each group. Next, the integrated gradient approach was used to assess the contribution of the features in reconstructing the original data, and the integrated gradient values of the control and autism groups were compared. Finally, we evaluated the associations between the symptom severity of autism, measured using the Autism Diagnostic Observation Schedule (ADOS), and the integrated gradient values.

## Method

### Study participants

We obtained T1-weighted and diffusion MRI data from three independent sites (New York University Langone Medical Center [NYU], Trinity College Dublin [TCD], and San Diego State University [SDSU]) of the Autism Brain Imaging Data Exchange-II (ABIDE-II) database [22]. We included the sites that (i) included children and adults with autism and neurotypical controls, with ≥ 10 individuals per group, (ii) who had T1-weighted and diffusion MRI available, (iii) sufficient MRI data quality (i.e., scanned with 3 T scanner). Of the 178 participants, 141 participants, including 61 neurotypical controls (mean ± standard deviation [SD] age = 13.2 ± 4.0 years) and 80 individuals with autism (12.1 ± 4.9 years), were included in the study (Table 1). Individuals with autism were diagnosed with ADOS [23] and/or Autism Diagnostic Interview-Revised [24], and the neurotypical controls did not have any history of mental illness. ABIDE data collection was performed in accordance with the local Institutional Review Board guidelines. In accordance with the Health Insurance Portability and Accountability Act (HIPAA) guidelines and 1000 Functional Connectomes Project/INDI protocols, all ABIDE datasets were fully anonymized, with no protected health information included.

**Table 1:** Demographic information of study participants

Information		NYU		TCD		SDSU		p-value^b^
Number (Autism/Control)		29/18		18/19		33/24		0.475^a^
Age	Autism	9.61 ± 6.16	*p* = 0.807	14.46 ± 3.30	*p* = 0.209	12.89 ± 3.23	*p* = 0.559	0.004
	Control	10.01 ± 3.95		15.83 ± 3.21		13.39 ± 2.96		0.003
Sex (male:female)	Autism	24:5	*p* = 0.243^a^	18:0	*p* = 1^a^	26:7	*p* = 0.343^a^	0.117^a^
	Control	17:1		19:0		22:2		0.450^a^
ADOS – Total		10.00 ± 3.36		8.72 ± 2.44		–		0.192
ADOS – Social cognition		7.50 ± 2.09		5.78 ± 2.37		–		0.023
ADOS – Communication		2.5 ± 1.70		2.94 ± 0.87		–		0.326
ADOS – Repeated behavior/interest		1.40 ± 1.27		0.22 ± 0.55		–		< 0.001

Mean and standard deviation are reported

*NYU* New York University Langone Medical Center, *TCD* Trinity College Dublin, *SDSU* San Diego State University, *ADOS* Autism Diagnostic Observation Schedule

^a^Chi-square test

^b^The p-values were reported for the lowest value among three possible combinations from three groups

### MRI data acquisition

T1-weighted and diffusion MRI from three independent sites, NYU, TCD, and SDSU, were scanned with 3 T Siemens Allegra, 3 T Philips Achieva, and 3 T GE MR7550 scanners, respectively. At the NYU site, T1-weighted images were acquired using a 3D magnetization-prepared rapid acquisition gradient echo (MPRAGE) sequence (repetition time [TR] = 2530 ms; echo time [TE] = 3.25 ms; inversion time [TI] = 1,100 ms; flip angle [FA] = 7˚; matrix size = 256 × 192; and voxel size = 1.3 × 1.0 × 1.3 mm^3^). Diffusion MRI data were obtained using a 2D spin-echo echo-planar imaging (SE-EPI) sequence (TR = 5200 ms; TE = 78 ms; matrix size = 64 × 64; voxel size = 3 mm^3^ isotropic; 64 directions; b-value = 1000 s/mm^2^; and 1 b0 image). At the TCD site, T1-weighted data were obtained using a 3D MPRAGE sequence (TR = 8,400 ms; TE = 3.90 ms; TI = 1,150 ms; FA = 8˚; matrix = 256 × 256; and voxel size = 0.9 mm^3^ isotropic). The diffusion MRI data were acquired using a 2D SE-EPI (TR = 20,244 ms; TE = 7.9 ms; matrix size = 124 × 124; voxel size = 1.94 × 1.94 × 2 mm^3^; 61 directions; b-value = 1,500 s/mm^2^; and 1 b0 image). At the SDSU site, the T1-weighted images were acquired using a 3D standard fast spoiled gradient echo (SPGR) sequence (TR = 8.136 ms; TE = 3.172 ms; TI = 600 ms; FA = 8˚; matrix size = 256 × 192; and voxel size = 1 mm^3^ isotropic). Diffusion MRI data were obtained using a 2D SE-EPI sequence (TR = 8,500 ms; TE = 84.9 ms; matrix size = 128 × 128; voxel size = 1.875 × 1.875 × 2 mm^3^; 61 directions; b-value = 1000 s/mm^2^; and 1 b0 image).

### MRI data preprocessing and structural connectivity construction

T1-weighted MRI data were preprocessed using a conventional recon-all process in FreeSurfer [25]. The process included gradient non-uniformity correction, non-brain tissue removal, intensity normalization, tissue segmentation, and surface reconstruction. The cortical surfaces were then topology corrected and inflated. Subsequently, a spherical registration to the fsaverage template space was performed. Diffusion MRI data were preprocessed using MRtrix3 [26], which corrected for susceptibility distortions, head motion, and eddy currents. Based on probabilistic tractography, we constructed structural connectomes from the preprocessed diffusion MRI data. Different tissue types, including cortical and subcortical gray matter, white matter, and cerebrospinal fluid, were defined from the T1-weighted data using anatomically-constrained tractography [27] and registered onto the native diffusion MRI space with boundary-based registration. Multishell and multitissue response functions were estimated [28], and constrained spherical deconvolution and intensity normalization were performed [29]. A tractogram was generated with 40 million streamlines, with a maximum tract length of 250, and a fractional anisotropy cutoff of 0.06. Then, spherical-deconvolution informed filtering of tractograms (SIFT2) was applied to reconstruct whole-brain streamlines weighted by cross-section multipliers [30]. The streamlines were mapped onto the Schaefer atlas with 200 parcels [31] and log-transformed to generate a structural connectivity matrix.

### Feature representation learning based on autoencoder

**Fig. 1:**
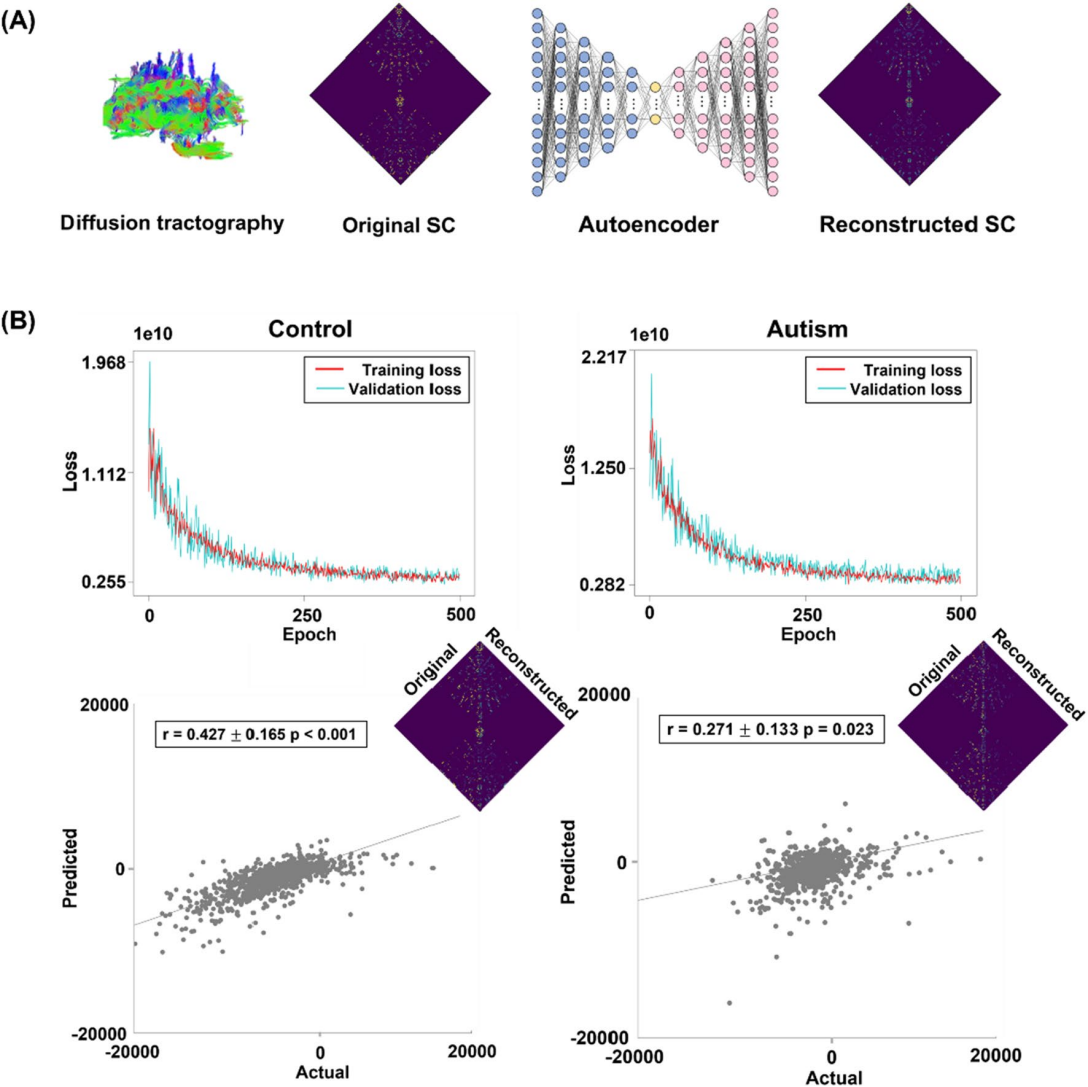
The autoencoder model of neurotypical controls and individuals with autism. **A** A schematic of the autoencoder model consisting of four encoder, one bottleneck, and four decoder layers. The diffusion tractography-based structural connectivity (SC) is entered into the autoencoder. **B** The upper panels show the loss graphs of training and validation datasets according to epochs for neurotypical controls (left) and individuals with autism (right). The scatter plots on the bottom represent correlations between the actual and reconstructed structural connectivity of the test data

We selected an autoencoder model to generate low-dimensional latent features from the input structural connectivity matrix [15, 16]. We controlled for age, sex, and site from the structural connectivity matrix using a linear regression model and entered the controlled data into the autoencoder by considering the left and right hemispheres separately. The autoencoder reconstructs the original data via encoding and decoding processes as follows:

1 $$z=E\left(x\right)={\text{tanh}}\left(Wx+b\right)$$

2 $$y=D\left(z\right)={\text{tanh}}\left(Wz+b\right)=D\left(E\left(x\right)\right)$$

3 $$L\left(x,y\right)=\sum \frac{{\left|x-y\right|}^{2}}{n}=\sum \frac{{\left|x-D\left(E\left(x\right)\right)\right|}^{2}}{n}$$

where *E* is the encoder that generates the latent feature *z* from the input data $x$, with weight $W$ and bias $b$. Then, the latent feature $z$ is entered into the decoder $D$ to reconstruct the input data and generate $y$. $L\left(x,y\right)$ is the loss function defined by the sum of the mean square errors between the input $x$ and output $y$, and the model is trained to minimize the $L\left(x,y\right)$. Our autoencoder model consists of four encoder layers, one bottleneck layer, and four decoder layers (Fig. 1A). The layers of the encoder and decoder had 7700; 5500; 2930; and 900 units, respectively, and the bottleneck layer contained 200 units. A dropout rate of 0.3 was applied to the input layer, and the hypertangent activation function was used for all layers. An average stochastic gradient descent optimizer [32] with a learning rate of 0.00008 was used. We constructed an autoencoder model for autism and neurotypical control groups, respectively. The data were randomly divided into the training (autism/control = 45 /33), validation (n = 20/16), and test (n = 15/12) datasets. Among the 500 epochs, we selected the weights that exhibited the minimum loss in the training and validation datasets and applied them to the test dataset. The performance of the model was assessed using the test dataset by calculating Pearson’s correlation between the original and reconstructed structural connectivity matrices. We repeated this process 100 times with different training, validation, and test datasets to minimize the subject selection bias. As the sensitivity analysis, we assessed the reconstruction performance of the autoencoder by changing the hyperparameters of the network architecture. First, we changed the number of units to (i) 7000; 5000; 2930; and 900, and (ii) 8000; 5000; 1000; and 500. Second, we changed the dropout rate to 0.1 and 0.5. Third, we constructed the architecture using five layers by including one additional layer with 4,400 units between layers 2 and 3. In addition, we constructed the architecture with three layers by removing layer 4. Lastly, we varied the learning rate to 0.0001 and 0.001.

### Contribution of latent features and between-group differences

We hypothesized that individuals with autism and neurotypical controls might show different reconstruction performances and that these differences may be associated with altered connectivity among different brain regions. We quantitatively assessed the extent of the contribution of structural connectivity while reconstructing the original data using the integrated gradient technique [21]. Briefly, it computes the attribution of each element of the structural connectivity matrix to predict the latent features of the bottleneck layer by progressively increasing the intensity of the input values from a zero-information baseline to a particular intact input level and averaging the attributions. The integrated gradient from ${i}^{{\text{th}}}$ neuron is defined as follows:

4 $$I{ntegrated \, Gradient }_{i}\left(x\right)=\left({x}_{i}-{m}_{i}\right)\times \underset{\alpha =0}{\overset{1}{\int }}\frac{\delta f\left(m+\alpha \left({x}-{m}\right)\right)}{\delta {x}_{i}}d\alpha,$$

where $x$ is the input, $m$ is the baseline, and $\alpha$ the interpolation constant. This can be represented by a summation using the Riemann approximation of the integral as follows:

5 $$I{ntegrated \, Gradient }_{i}\left(x\right)=\left({x}_{i}-{m}_{i}\right)\times \frac{1}{M}{\sum }_{k=1}^{M}\frac{\delta f\left(m+\frac{k}{M}\left({x}-m\right)\right)}{\delta {x}_{i}},$$

where *m* and *M* are the number of steps in the scaled feature perturbation constant and the approximation of the integral, respectively. Thus, it provides information on how each element of the connectivity matrix contributes significantly to the encoding processes. We compared the integrated gradient values of each individual between individuals with autism and neurotypical controls after the z-normalization of these values. Between-group differences were assessed using two-sample t-tests with 1000 permutation tests by randomly assigning group indices. A null distribution was constructed, and if the real t-statistic did not belong to the 5% of the null distribution, it was considered significant. The p-values were corrected using a false discovery rate (FDR) < 0.05 [33]. To assess network-level differences, we summarized the t-statistic values based on seven intrinsic functional communities as follows: visual, somatomotor, dorsal attention, ventral attention, limbic, frontoparietal, and default mode networks [34]. We additionally assessed the effects of between-group differences using the structural connectivity instead of the integrated gradient values between neurotypical controls and individuals with autism.

### Symptom severity associations

To determine whether the integrated gradient values were associated with the symptom severity of autism measured by the ADOS, which included the social cognition, communication, and repeated behavior/interest sub-scores, as well as the total score [3], we adopted canonical correlation analysis (CCA) [35]. CCA finds a canonical coordinate space that maximizes the correlations between the projections of different datasets onto the space [36]. Here, we projected the integrated gradient values (**X**) and ADOS scores (**Y**) onto each dimension of the canonical space and obtained the canonical components **u** and **v** such that these components had maximum correlations. The optimal number of canonical components was determined using a five-fold cross-validation. For each cross-validation, we selected components that showed significant (FDR < 0.05) correlations between **u** and **v**. The explained variance assesses how much of the variance of the data is explained by each component and is defined as follows:

$$Explained \, Variance \left(x\right)= \frac{Var\left\{x\right\}-Var\{x- \widehat{x}\}}{Var\{x\}},$$

where *x* is the original data, and $\widehat{x}$ is the predicted data.

Additionally, we estimated the explained variance of each component and summarized the explained variance of the integrated gradients within and between the networks using seven functional communities [34]. Furthermore, we assessed the multivariate associations between the ADOS scores and structural connectivity data or the latent vectors extracted from the bottleneck layer of the autoencoder to evaluate which feature is more useful for explaining the symptoms of autism. The symptom severity association analysis was performed using the data from NYU and TCD sites because the SDSU site did not provide the ADOS score.

## Result

### The autoencoder model and reconstruction performance

The structural connectivity matrix was entered into an autoencoder and trained to reconstruct the original data (Fig. 1A). We observed decreasing loss values in both the training and validation datasets across epochs (Fig. 1B). The weights of the latest epoch that exhibited the best performance were applied to the test dataset. The reconstruction performance based on the correlations between the original and reconstructed connectivity matrix was significant for control (mean ± SD across individuals and 100 bootstraps, r = 0.427 ± 0.165, p < 0.001) and autism groups (autism: r = 0.271 ± 0.133, p = 0.023; Fig. 1B). When we tested the reconstruction performance with different settings of hyperparameters, the performances were lower than our model (Additional file 1: Table S1).

### Between-group differences in the integrated gradients

**Fig. 2:**
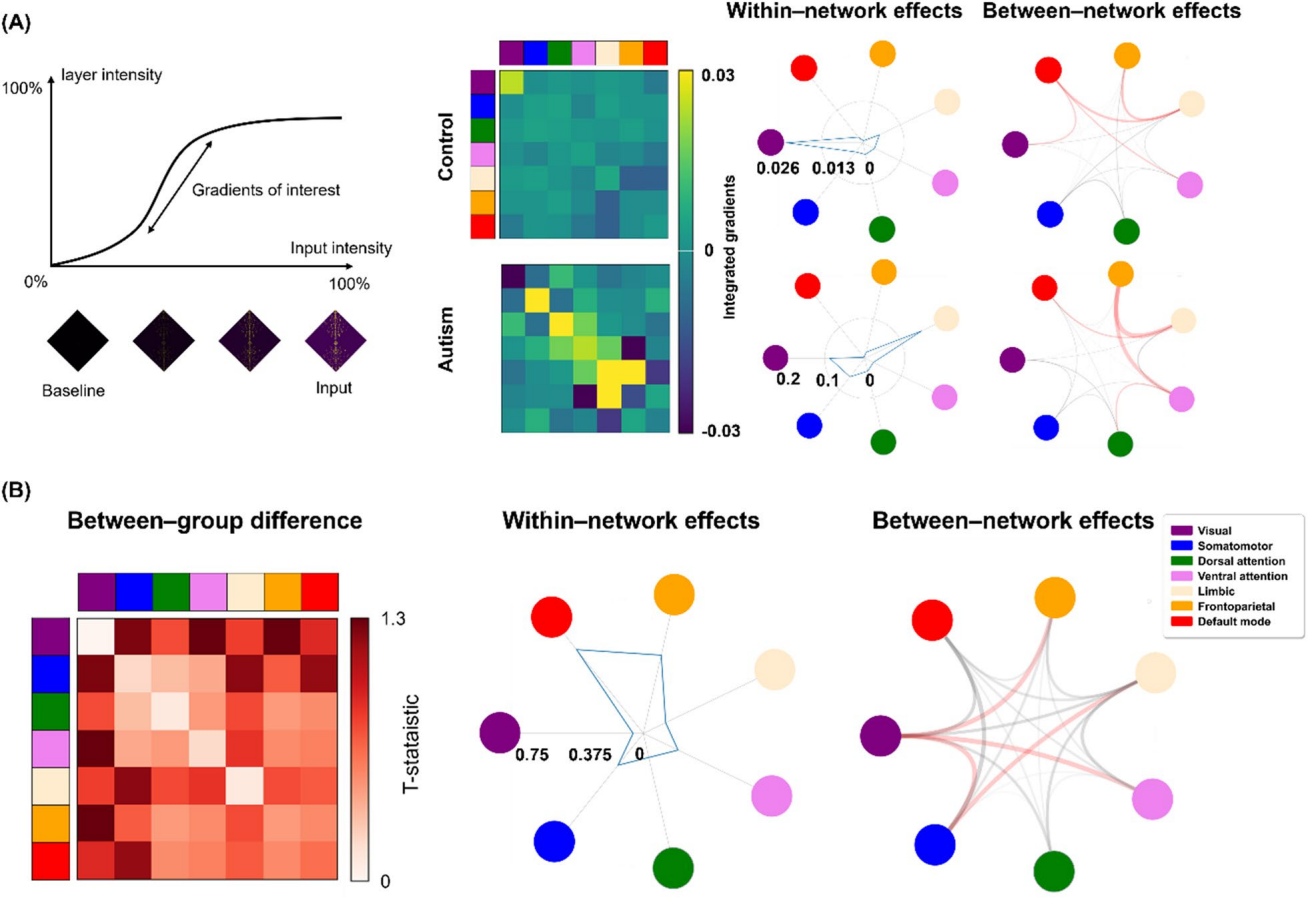
Between-group differences in the integrated gradient values. **A** Shown is the schema of the integrated gradient technique (left). We summarized the integrated gradient values of neurotypical controls and individuals with autism based on seven functional communities (right). **B** We compared the integrated gradient values between individual with autism and neurotypical controls after the z-normalization. We assessed two-sample t-tests with 1000 permutation tests by shuffling group indices. We then applied a false discovery rate (FDR) < 0.05. The t-statistics of the between-group differences are shown (left). Within- and between-network effects are plotted based on the t-statistics of the between-group differences (right). The highest effects of between-network were shown in red lines. The high effects were observed within the default mode and frontoparietal networks, and between the visual and frontoparietal/ventral attention networks as well as between the somatomotor and limbic networks

The integrated gradient method was applied to assess the contribution of the structural connectivity to predict latent features of the hidden layer (Fig. 2A). We found that within the visual network and between the limbic and frontoparietal networks showed particularly large attributions in neurotypical controls, while within the limbic network and between the limbic and frontoparietal networks showed large effects in individuals with autism. We assessed between-group differences in the integrated gradient values and summarized the t-statistic values of the connections that showed significant between-group differences according to the seven functional communities [34]. We observed high effects within the default mode and frontoparietal networks, and between the visual and frontoparietal/ventral attention networks as well as between the somatomotor and limbic networks (Fig. 2B). The small effects were observed within the visual network and between the somatomotor and dorsal attention networks. Furthermore, we examined between-group differences in the structural connectivity between neurotypical controls and individuals with autism, and only two elements (visual-default mode and frontoparietal-default mode networks) showed significant effects, suggesting lower sensitivity of the structural connectivity in assessing connectome distortions in individuals with autism than integrated gradient values.

### Associations between symptom severity and the integrated gradients

**Fig. 3:**
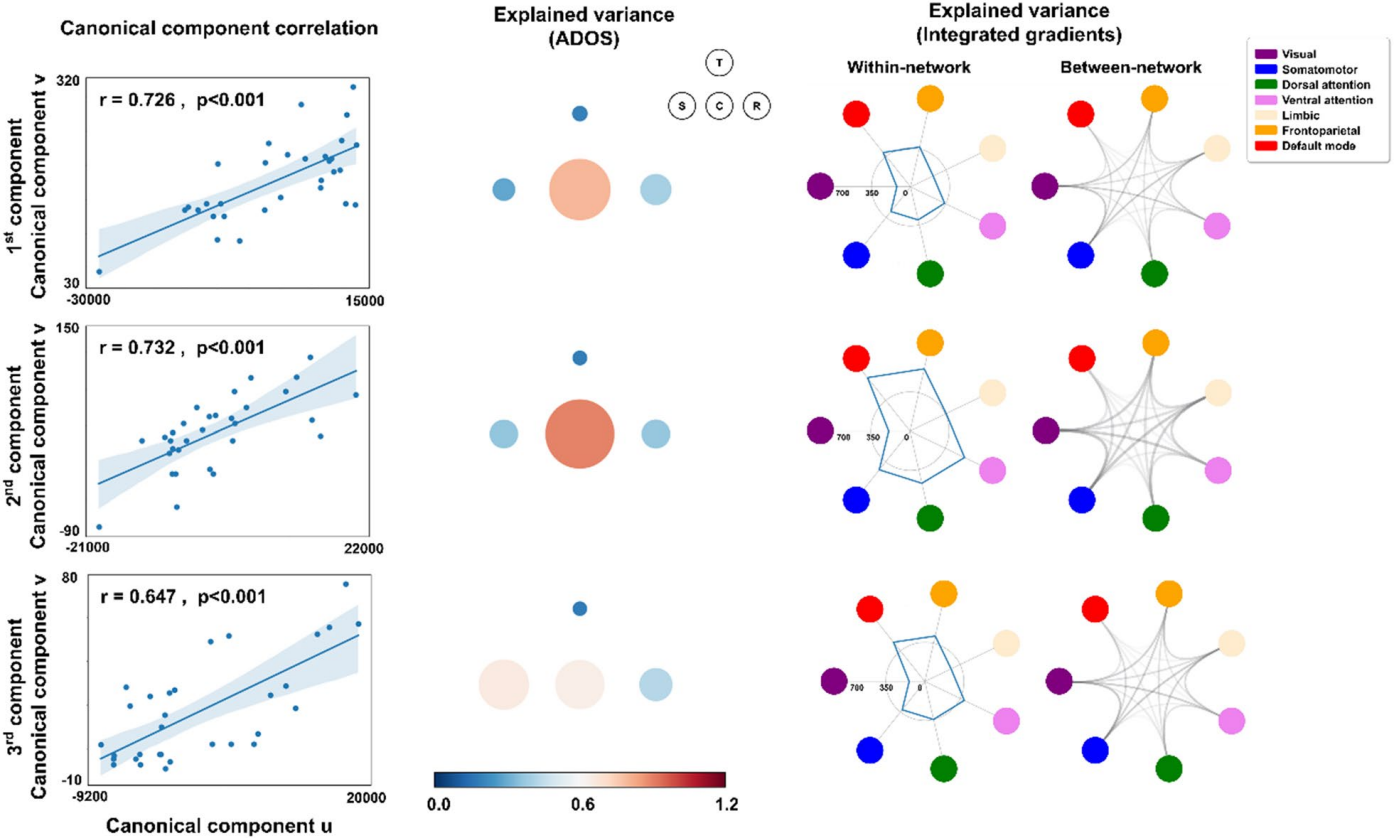
Canonical correlation analysis between symptom severity of autism and the integrated gradient values. Correlations between the canonical components **u** and **v** (left) are shown. Circle plots represent the explained variance of ADOS total, social cognition, communication, and repeated behavior/interest scores (middle). The size and color of the circles indicate the magnitude of the explained variance of each ADOS score. The explained variance of the integrated gradients of within- and between-networks is shown with spider plots (right). The ADOS communication sub-score, within the default mode network, and between the somatomotor-visual/limbic/frontoparietal networks showed relatively high explained variance. *Abbreviations*: ADOS, Autism Diagnostic Observation Schedule; T, total; S, social cognition; C, communication; R, repeated behavior/interest

We assessed the associations between the integrated gradient values and symptom severity of autism measured by the ADOS [23] using CCA [35]. Five-fold cross-validation showed that the optimal number of canonical components was three. Each canonical component showed significant correlations (1st: r = 0.726, p < 0.001; 2nd: r = 0.732, p < 0.001; 3rd: r = 0.647, p < 0.001; Fig. 3). When we estimated the explained variance, the ADOS communication sub-score was associated with the integrated gradient values within the default mode network and between the somatomotor-visual/limbic/frontoparietal networks. These findings suggest that sensory and transmodal (i.e., default mode and frontoparietal) regions may be critically associated with communication skills in autism. When we conducted CCA using structural connectivity, the correlations between the canonical components and ADOS scores showed lower performance than when we used the integrated gradient values. In addition, the correlations based on the latent vectors did not show significant associations (Additional file 1: Table S2). Together, the findings indicate that the CCA analysis based on the integrated gradient values is more beneficial than using structural connectivity or latent vectors.

## Discussion

Understanding whole-brain structural connectome disorganization in individuals with autism may complement previous functional findings; however, studies identifying principal markers related to autism pathophysiology are relatively underinvestigated owing to the lack of interpretability of deep learning techniques. In this study, we systematically investigated the structural connectome abnormalities in individuals with autism using feature representation learning combined with an integrated gradient approach. We observed that the low-dimensional features of structural connectivity within the transmodal regions, including the default mode and frontoparietal networks, and between the sensory and limbic systems, were altered in individuals with autism. Additionally, we found that these features were associated with the communication abilities of individuals with autism, suggesting their clinical implications. Our findings provide an understanding of the atypical structural connectivity in individuals with autism and its association with their clinical phenotypes.

The core of our study is feature representation learning, which generates low-dimensional latent features [15, 16]. In neuroscience, the gradient approach, which estimates low-dimensional eigenvectors from connectome data, is widely adopted to investigate the whole-brain connectome organization of brain structure and function [12, 37–43] and their relations [44–46]. However, these studies were primarily based on nodal-level analysis and did not consider the interconnected links between nodes. To fill this gap, we generated latent vectors from the connectivity matrix using an autoencoder model to assess edge-level effects. However, a crucial point to consider when using deep learning-based models is the uncertainty of interpretation. Here, we opted for the integrated gradient method, which calculates the contribution of the input features in predicting the latent vectors [21]. We found that feature contributions differed between individuals with autism and neurotypical controls, particularly in connectivity within the transmodal regions and between the sensory and limbic networks. Sensorimotor and default mode networks show connectome idiosyncrasies and a decrease in the number of neurotransmitter receptors in individuals with autism [3, 4, 47]. In addition, abnormal structural connectomes in these systems are associated with excitation/inhibition imbalances in autism [5]. Our work expands upon prior studies by providing insights into the understanding of low-dimensional representations of structural connectivity in autism.

To assess the behavioral associations of the low-dimensional features of whole-brain structural connectivity in individuals with autism, we used a multivariate association technique called CCA. Unlike conventional association analyses based on linear correlations or regression analyses, CCA determines the canonical coordinate space that maximizes the correlation between independent and dependent variables. We found that the integrated gradient values within the default mode region and between the sensory and transmodal regions were highly associated with communication skills in individuals with autism. The sensory and transmodal regions are involved in the perception and processing of language and nonverbal information [48, 49]. Individuals with autism show decreased activation in the inferior frontal cortex [50], anterior insula, and premotor cortex [51], and these patterns are associated with alterations in social information integration [52]. Behavioral studies have also found that individuals with autism show lower verbal and nonverbal abilities than neurotypical controls [53, 54]. Together, these studies suggest that altered connectome organization in sensory and transmodal areas may be related to communication and social impairments in individuals with autism [55], and that the integrated gradient values derived from the autoencoder model could serve as an indicator for describing the symptom severity of autism.

In this study, we identified structural connectivity differences in individuals with autism using feature representation learning combined with integrated gradient techniques, which may provide potential biomarkers for the diagnosis of autism. However, this study has several limitations. First, our study had a small sample size owing to the nature of the ABIDE database and strict quality control procedures. In the future, additional data must be collected from independent databases to improve the reliability of our findings. Second, we used the integrated gradient method to interpret the internal operations of the autoencoder. In future studies, we need to link integrated gradient data to biologically plausible mechanisms for better neuroscientific insights. Finally, the estimated integrated gradient values may be variable according to the baseline. Although the zero-informed baseline is commonly used for the image data, it should be noted that the different baselines may yield varying results and thus need careful interpretation.

### Supplementary Information

**Additional file 1: Table S1.** Reconstruction performance of the autoencoder with different hyperparameter settings. The performance was assessed by calculating Pearson’s correlation between the actual and reconstructed structural connectivity of the test data. **Table S2.** Correlations between the canonical components of the ADOS scores and various features.

## Data Availability

Imaging and phenotypic data were provided, in part, by the Autism Brain Imaging Data Exchange initiative (ABIDE-II; https://fcon_1000.projects.nitrc.org/indi/abide). Codes for the autoencoder and integrated gradients are available at https://github.com/CAMIN-neuro/caminopen/tree/master/Feature_representation_learning_autism.
