# Supplementary material for: Structural connectome alterations between individuals with autism and neurotypical controls using feature representation learning
Source: Behav Brain Funct. 2024 Jan 24;20:2. doi: 10.1186/s12993-024-00228-z (PMC10807082; doi:10.1186/s12993-024-00228-z)
Supplement: Supplementary file 1 — Additional file 1: Table S1. Reconstruction performance of the autoencoder with different hyperparameter settings. The performance was assessed by calculating Pearson’s correlation between the actual and reconstructed structural connectivity of the test data. Table S2. Correlations between the canonical components of the ADOS scores and various features. [file 12993_2024_228_MOESM1_ESM.docx]

**Additional file 1**

**Table S1 | Reconstruction performance of the autoencoder with different hyperparameter settings.** The performance was assessed by calculating Pearson’s correlation between the actual and reconstructed structural connectivity of the test data.

| **Parameter settings** | | **Control** | **Autism** |
| --- | --- | --- | --- |
| Ours  - Unit parameters: 7,700; 5,500; 2,930; 900  - Dropout rate: 0.3  - Number of layers: 4  - Learning rate: 0.00008 | | r = 0.427 \| p = 0.0016 | r = 0.271 \| p = 0.023 |
| Number of units | 7,000; 5,000; 2,930; 900 | r = 0.309 \| p < 0.001 | r = 0.278 \| p < 0.001 |
|  | 8,000; 5,000; 1,000; 500 | r = 0.372 \| p < 0.001 | r = 0.158 \| p = 0.0017 |
| Dropout rate | 0.1 | r = 0.157 \| p < 0.001 | r = 0.112 \| p < 0.001 |
|  | 0.5 | r = 0.203 \| p < 0.001 | r = - 0.001 \| p = 0.018 |
| Number of layers | 5 | r = 0.354 \| p < 0.001 | r = 0.158 \| p < 0.001 |
|  | 3 | r = 0.225 \| p = 0.012 | r = 0.192 \| p < 0.001 |
| Learning rate | 0.0001 | r = 0.151 \| p < 0.001 | r = 0.152 \| p = 0.184 |
|  | 0.001 | r = -0.108 \| p < 0.001 | r = 0.081 \| p < 0.001 |

**Table S2 | Correlations** **between the canonical components of the ADOS scores and various features.**

| **Features** | **1st component** | **2nd component** | **3rd component** |
| --- | --- | --- | --- |
| Integrated gradients | r = 0.726 \| p < 0.001 | r = 0.732 \| p < 0.001 | r = 0.647 \| p < 0.001 |
| Structural connectivity | r = 0.561 \| p < 0.001 | r = 0.484 \| p = 0.008 | r = 0.434 \| p = 0.021 |
| Latent vector | r = 0.193 \| p = 0.922 | r = 0.031 \| p = 0.926 | r = 0.002 \| p = 0.940 |
